# Factors associated with underutilization of antenatal care in India: Results from 2019–2021 National Family Health Survey

**DOI:** 10.1371/journal.pone.0285454

**Published:** 2023-05-08

**Authors:** Nandan Thakkar, Prima Alam, Deepak Saxena

**Affiliations:** 1 Office of Graduate Education, University of North Carolina School of Medicine, Chapel Hill, NC, United States of America; 2 Public Health and Policy, London School of Hygiene & Tropical Medicine, London, United Kingdom; 3 Indian Institute of Public Health—Gandhinagar, Gandhinagar, Gujarat, India; Jawaharlal Nehru Medical College, INDIA

## Abstract

**Introduction:**

Despite progress in recent years, full antenatal care utilization in India continues to be relatively low and inequitable, particularly between states and districts. In 2015–2016, for example, only 51% of women aged 15–49 in India attended antenatal care at least four times during pregnancy. Using data from the fifth iteration of India’s National Family Health Survey, our study aims to explore factors related to the underutilization of antenatal care in India.

**Materials and methods:**

Data from the most recent live birth in the past five years among women aged 15–49 years were included in our analysis (n = 172,702). Our outcome variable was “adequate antenatal care visits”, defined as four or more antenatal visits. Utilizing Andersen’s behavioral model, 14 factors were identified as possible explanatory variables. We used univariate and multivariate binary logistic regression models to analyze the association between explanatory variables and adequate visits. Associations were considered statistically significant if p<0.05.

**Results:**

Of the 172,702 women in our sample, 40.75% (95% CI: 40.31–41.18%) had an inadequate number of antenatal care visits. In multivariate analysis, women with less formal education, from poorer households and more rural areas had higher odds of inadequate visits. Regionally, women from Northeastern and Central states had higher odds of inadequate antenatal care utilization compared to those from Southern states. Caste, birth order, and pregnancy intention were also among the variables associated with utilization of antenatal care.

**Discussion:**

Despite improvements in antenatal care utilization, there is cause for concern. Notably, the percentage of Indian women receiving adequate antenatal care visits is still below the global average. Our analysis also reveals a continuity in the groups of women at highest risk for inadequate visits, which may be due to structural drivers of inequality in healthcare access. To improve maternal health and access to antenatal care services, interventions aimed at poverty alleviation, infrastructure development, and education should be pursued.

## Introduction

Over the past two decades, India has seen a 70% fall in overall maternal mortality ratio (MMR) from 398/100,000 live births to 99/100,000 live births in 2020 [1]. As such, India is well on track towards achieving maternal mortality targets set by the United Nations Sustainable Development Goals (SDGs), which aims to reduce global maternal mortality to less than 70/100,000 live births by 2030 with no single country having an MMR of more than 140/100,000 live births [2]. Despite this progress, the country still accounted for 12% of all global maternal deaths, second only to Nigeria [3]. Over 60% of India’s 23,800 maternal deaths in 2020 occurred in poorer states such as Assam and Uttarakhand [1]. As with maternal mortality in other low- and middle-income countries (LMICs)–which account for almost 99% of global maternal deaths–many of these deaths are preventable with greater access to quality healthcare and effective interventions during the preconception, antenatal, intrapartum, and post-natal periods [4–8].

Access to high-quality maternal health care before, during, and after childbirth has been identified as an effective way of reducing preventable maternal deaths [3, 9, 10]. Antenatal care (ANC) provides a crucial opportunity for skilled healthcare professionals to address potential health risks during pregnancy through disease prevention, identification, and management [11, 12]. ANC also allows providers to engage pregnant women in both immediate and long-term health promotion and education [9, 13]. The overall goal of ANC is to provide care that ensures the best health conditions for both mother and baby during pregnancy [11].

Recognizing the life-saving potential of sufficient antenatal care, the World Health Organization (WHO) has issued guidelines for the provision of adequate care during pregnancy, which highlight established interventions known to improve maternal and neonatal health outcomes [11, 14]. Introduced in 2002, WHO’s Focused Antenatal Care model recommended pregnant women receive at least four comprehensive antenatal care visits with a qualified healthcare provider during pregnancy [14]. Ideally, the first visit should be scheduled during the first trimester of pregnancy, the second visit during the second trimester, and the third and fourth visits during the third trimester. Furthermore, to ensure SDGs addressing maternal mortality are met by 2030, the World Health Organization has also set a global coverage target of 90% pregnant women attending four or more antenatal care visits by 2025 [15].

Since their inception in 1992, India’s national health surveys have recorded consistent positive trends in sufficient antenatal care utilization among pregnant women, with a greater portion of population reporting at least four ANC visits with a healthcare provider [16]. Despite positive trends, according to the 2015–16 National Family Health Survey (NFHS), only 51% of women aged 15–49 attended at least four ANC visits. Additionally, only one out of four women in India received adequate antenatal care [17]. As with the reduction in maternal deaths, full antenatal care utilization in India has been inequitable, particularly between states and districts [18–20]. Factors such as caste, maternal education, and family income have also contributed to disparities in utilization [9, 19].

**Fig 1:**
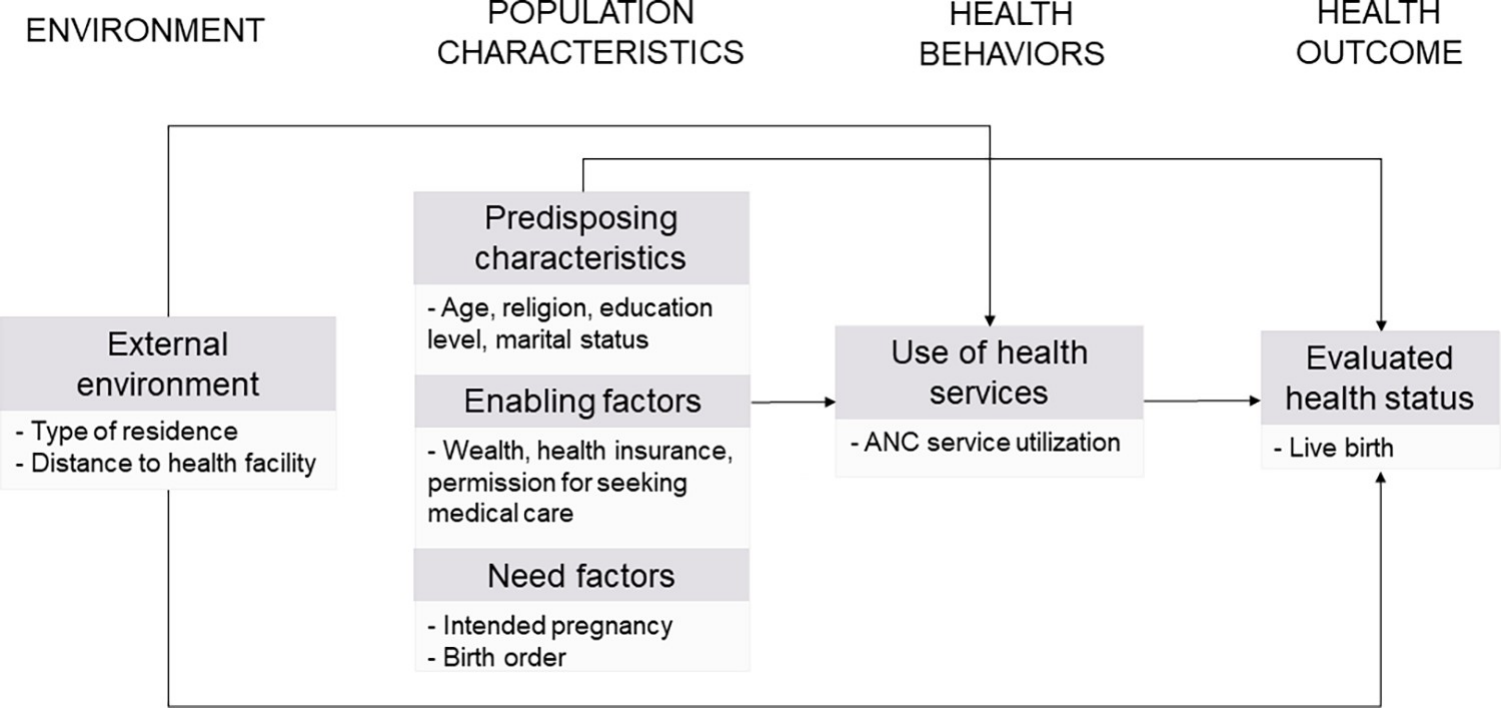
Conceptual framework of factors associated with utilization of antenatal care*. * Conceptual framework adapted from Andersen’s behavioral model of health service use [21] as well as ANC-specific studies in Nepal [22] Indonesia [23].

By adapting Andersen’s behavioral model of health service use, our study aims to explore factors related to the underutilization of antenatal care in India [21]. As Fig 1 shows, the conceptual framework helps us to identify and organize our analysis around external environmental factors, predisposing characteristics, enabling factors, and need factors. For the purpose of this article, we define underutilization of antenatal care–or inadequate antenatal care–as fewer than four antenatal care visits provided by skilled healthcare professionals [11]. While there have been previous studies investigating determinants of antenatal care use in India, there is a need to engage with up-to-date data to determine current gaps in antenatal care coverage and utilization. Therefore, we use data from the 2019–2021 Demographic and Health Survey (DHS)–also known as the National Family Health Survey–to explore which factors are contributing to the underutilization of antenatal care in India. Findings from this article can be used to inform policymakers and healthcare practitioners of major barriers to full antenatal utilization and facilitate the design of interventions to address these barriers.

## Materials and methods

### Data source

Our cross-sectional study analyzed data from the fifth iteration of India’s National Family Health Survey (NFHS-5), also known as the 2019–2021 Demographic Health Survey [24]. Coordinated by the International Institute for Population Sciences (IIPS), the NFHS is a multi-round survey conducted in a representative sample of 639,699 households throughout India [24]. Using a standardized questionnaire, 724,115 eligible woman aged 15–49 years were interviewed. Information on several topics–including basic sociodemographic characteristics, maternal health care, reproductive behavior–was collected. Additional survey details regarding sampling methodology and survey design can be found in India’s official NFHS-5 report as well as prior studies [24].

Original women’s data were reformatted using common variable names to generate the Individual Recode dataset (IAIR7CFL), which the authors accessed for this study. Weighted data from women aged 15–49 years with a live birth in the past five years were included in our analysis (N = 174,947). Respondents with missing data and those unable to recall information regarding ANC visits were excluded (n = 2,245) for a final sample of 172,702 women.

This study was a secondary analysis of de-identified data from the NFHS dataset for India, which is readily available in the public domain. Informed consent was obtained from original survey participants and survey protocol was approved by institutional review board at the International Institute for Population Sciences, Mumbai.

### Outcome and explanatory variables

Our outcome variable was “adequate antenatal care visits”, defined as four or more antenatal visits as per WHO standards [11]. For our analysis, ANC visits were categorized to produce a dichotomous dependent variable with the following outcomes: adequate ANC (≥4 visits) and inadequate ANC (<4 visits). Visits with health professionals such as doctors, nurses, midwives, and skilled birth attendants were considered. For women with multiple live births in the five-year data collection period, only information from the most recent pregnancy was analyzed.

Utilizing the Andersen behavioral conceptual framework, pertinent ANC research, and a priori understanding; we identified the following 14 factors as explanatory variables to consider in our analysis: place of residence; region; maternal age; religion; caste; marital status; maternal education level; household wealth index; pregnancy intention; birth order; health insurance coverage; history of miscarriage, abortion or stillbirth; ability to receive permission to seek medical care; ability to get money for medical treatment; and distance to health facility [10, 21–23]. Consistent with Andersen’s framework, these factors were further grouped into the following categories: external environment, predisposing characteristics, enabling factors, and need factors (see Fig 1).

### Statistical analysis

Our statistical approach was similar to previous published research [10, 19, 23]. Initially, a descriptive analysis was conducted to report survey frequencies and prevalence of adequate antenatal care visits by study factor. We then used binary logistic regression to analyze the association between explanatory variables and adequate antenatal care visits. Variables with a p-value of <0.20 in the preliminary univariate analysis were included in the final multivariate model. We considered associations in the multivariate logistic regression analysis as statistically significant if the p-value was <0.05. Results are presented as crude (cOR) and adjusted odds ratios (aOR) with 95% confidence intervals (CI).

Our analysis was performed using STATA© 17.0 (StataCorp LLC, College Station, TX, USA). We used Stata’s *svyset* command to account for sampling weights, clustering, and stratification given the survey’s multi-stage sampling design. The authors acquired permission to access and analyze data from the DHS program prior to study commencement. Program guidelines for data use were also followed.

## Results

### Distribution of ANC utilization

Of the 172,702 women in our weighted sample, 40.75% (95% CI: 40.31–41.18%) had an inadequate number of antenatal care visits (Table 1). The prevalence of inadequate ANC visits was highest among those reporting a birth order of 6 or greater (68.17%); those lacking any formal education (59.80%); or those belonging to the poorest quintile (57.69%) (Table 2). Women who belonged to the wealthiest quintile (72.91%), attended higher education (72.15%), or lived in an urban setting (69.61%) had the highest prevalence of adequate ANC visits (Table 2).

**Table 1:** Frequency and prevalence of ANC utilization, NFHS-5.

Variable		N	Prevalence Rate [95% CI]
*Number of ANC visits*			
	≥4 (adequate)	102,334	59.25 [58.82–59.69]
	<4 (inadequate)	70,368	40.75 [40.31–41.18]

**Table 2:** Distribution of ANC utilization & characteristics of women who gave birth in last 5 years, NFHS-5.

	Variable		N	≥4 ANC N (%)	<4 ANC N (%)
**External environment**	*Place of residence *				
		Urban	48,595	33,826 (69.61)	14,770 (30.39)
		Rural	124,107	68,509 (55.20)	55,599 (44.80)
	*Region*				
		North	23,534	14,628 (62.16)	8,906 (37.84)
		Central	46,383	22,009 (47.45)	24,375 (52.55)
		East	44,549	22,374 (50.22)	22,175 (49.78)
		Northeast	6,939	3,683 (53.09)	3,255 (46.91)
		West	22,017	16,627 (75.52)	5,390 (24.48)
		South	29,280	23,013 (78.59)	6,267 (21.41)
**Predisposing characteristics**	*Maternal age*				
		15–19	5,370	3,276 (61.00)	2,094 (39.00)
		20–24	50,497	29,518 (58.46)	20,979 (41.54)
		25–29	67,026	40,058 (59.76)	26,968 (40.24)
		30–34	33,872	20,583 (60.77)	13,289 (39.23)
		35–39	12,469	7,116 (57.07)	5,352 (42.93)
		40–44	2,814	1,510 (53.66)	1,304 (46.34)
		45–49	655	273 (41.65)	382 (58.35)
	*Religion *				
		Hindu	137,703	81,502 (59.19)	56,201
		Muslim	27,347	15,870 (58.03)	11,477 (41.97)
		Christian	3,552	2,402 (67.61)	1,151 (32.39)
		Sikh	2,252	1,384 (61.47)	868 (38.53)
		Neo-Buddhist	861	580 (67.47)	280 (32.58)
		Others	988	596 (60.36)	392 (39.64)
	*Caste*				
		Scheduled Caste	39,199	21,921 (55.92)	17,278 (44.08)
		Scheduled Tribe	17,062	9,968 (58.42)	7095 (41.58)
		Other Backward Class	74,553	43,001 (57.68)	31,552 (42.32)
		None	40,377	26,540 (65.73)	13,837 (34.27)
		Don’t know	1,510	904 (59.82)	607 (40.18)
	*Education level (highest attended)*				
		No education	33,866	13,615 (40.20)	20,251 (59.80)
		Primary	20,308	10,918 (53.76)	9390 (46.24)
		Secondary	88,754	56,319 (63.46)	32,435 (36.54)
		Higher	29,774	21,482 (72.15)	8,292 (27.85)
	*Marital status *				
		Never married	160	75 (46.72)	85 (53.28)
		Currently married	170,545	101,129 (59.30)	69,416 (40.70)
		Widowed	1,074	576 (53.63)	498 (46.37)
		Divorced/Separated	923	554 (60.08)	368 (39.92)
**Enabling factors**	*Household wealth index*				
		Poorest	39,364	16,654 (42.31)	22,710 (57.69)
		Poorer	36,382	19,706 (54.17)	16,676 (45.83)
		Middle	33,841	21,428 (63.32)	12,413 (36.68)
		Richer	33,172	22,714 (68.47)	10,458 (31.53)
		Richest	29,943	21,831 (72.91)	8,111 (27.09)
	*Health insurance coverage*				
		Covered	41,229	26,658 (64.66)	14,571 (35.34)
		Not covered	131,472	75,676 (57.56)	55,797 (42.44)
	*Distance to health facility*				
		No problem [ref]	70,661	46,039 (65.15)	24,623 (34.85)
		Big problem	42,058	22,104 (52.56)	19,954 (47.44)
		Not a big problem	59,983	34,192 (57.00)	25,792 (43.00)
	*Getting money for treatment *				
		No problem [ref]	81,418	51,489 (63.24)	29,930 (36.76)
		Big problem	38,521	21,063 (54.68)	17,458 (45.32)
		Not a big problem	52,763	29,782 (56.45)	22,980 (43.55)
	*Receiving permission to seek medical help*				
		No problem	106,148	67,008 (63.13)	39,139 (36.87)
		Big problem	26,980	13,335 (49.43)	13,645 (50.57)
		Not a big problem	39,574	21,991 (55.57)	17,584 (44.43)
**Need factors**	*Pregnancy intention*				
		Wanted	158,910	95,544 (60.12)	63,367 (39.88)
		Not wanted/Wanted later	13,792	6,791 (49.24)	7,001 (50.76)
	*Birth order *				
		1st	59,086	39,168 (66.29)	19,919 (33.71)
		2nd– 3rd	91,190	54,029 (59.25)	37,161 (40.75)
		4th– 5th	18,026	7,737 (42.92)	10,289 (57.08)
		>6th	4,399	1,400 (31.83)	2,999 (68.17)

a: Regions defined by NFHS-5 report [25].

### Factors associated with ANC utilization from multivariate analysis

Results from univariate and multivariate analyses are presented in Table 3. Crude and adjusted odds ratios for ANC underutilization are included for each explanatory variable with relevant reference groups, significance levels, and confidence intervals. Study factors are also organized into categories as outlined by our adapted Andersen conceptual framework (Fig 1).

**Table 3:** Multiple logistic regression of factors associated with underutilization of ANC visits in India, NFHS-5.

	Variable		cOR*	p-value	95% CI	aOR**	p-value	95% CI
**External Environment**	*Place of residence*							
		Urban [ref]	1	---	---	1	---	---
		Rural	1.86	<0.001	1.82–1.90	1.12	<0.001	1.06–1.18
	*Region*							
		North	2.24	<0.001	2.09–2.39	2.15	<0.001	2.00–2.32
		Central	4.07	<0.001	3.81–4.34	2.92	<0.001	2.73–3.12
		East	3.64	<0.001	3.41–3.89	2.53	<0.001	2.36–2.72
		Northeast	3.24	<0.001	3.00–3.51	3.06	<0.001	2.81–3.34
		West	1.19	<0.001	1.08–1.31	1.10	0.058	1.00–1.22
		South [ref]	1	---	---	1	---	---
**Predisposing characteristics**	*Maternal age*							
		15–19 [ref]	1	---	---	1	---	---
		20–24	1.11	<0.001	1.05–1.18	1.07	0.137	0.98–1.17
		25–29	1.05	0.075	0.99–1.11	0.95	0.307	0.87–1.04
		30–34	1.01	0.743	0.95–1.07	0.82	<0.001	0.75–0.91
		35–39	1.18	<0.001	1.10–1.26	0.83	<0.001	0.72–0.90
		40–44	1.35	<0.001	1.23–1.48	0.73	<0.001	0.61–0.80
		45–49	2.19	<0.001	1.86–2.58	0.89	0.408	0.65–1.19
	*Religion *							
		Hindu	1.44	<0.001	1.34–1.55	0.99	0.898	0.88–1.11
		Muslim	1.51	<0.001	1.40–1.63	0.96	0.475	0.84–1.08
		Christian [ref]	1	---	---	1	---	---
		Sikh	1.31	<0.001	1.17–1.46	1.15	0.111	0.97–1.38
		Neo-Buddhist	1.01	0.917	0.86–1.18	1.31	0.161	0.90–1.90
		Others	1.37	<0.001	1.19–1.59	1.00	0.972	0.82–1.22
	*Caste*							
		Scheduled Caste	1.51	<0.001	1.47–1.56	1.19	<0.001	1.13–1.26
		Scheduled Tribe	1.37	<0.001	1.32–1.42	0.94	0.075	0.88–1.01
		Other Backward Class	1.40	<0.001	1.37–1.44	1.30	<0.001	1.24–1.37
		None [ref]	1	---	---	1	---	---
		Don’t know	1.29	<0.001	1.16–1.43	1.00	0.959	0.82–1.20
	*Education level (highest attended)*							
		No education	3.85	<0.001	3.73–3.98	1.76	<0.001	1.65–1.88
		Primary	2.23	<0.001	2.15–2.31	1.21	<0.001	1.13–1.29
		Secondary	1.49	<0.001	1.45–1.54	1.07	0.023	1.01–1.13
		Higher [ref]	1	---	---	1	---	---
	*Marital status *							
		Never married	1.66	0.001	1.22–2.27	1.59	0.075	0.95–2.65
		Currently married [ref]	1	---	---	1	---	---
		Widowed	1.26	<0.001	1.12–1.42	1.17	0.115	0.96–1.42
		Divorced/Separated	0.97	0.630	0.85–1.10	1.12	0.307	0.90–1.38
**Enabling factors**	*Household wealth index*							
		Poorest	3.67	<0.001	3.55–3.79	1.69	<0.001	1.57–1.81
		Poorer	2.28	<0.001	2.20–2.35	1.33	<0.001	1.24–1.42
		Middle	1.56	<0.001	1.51–1.61	1.16	<0.001	1.09–1.23
		Richer	1.24	<0.001	1.20–1.28	1.09	0.011	1.02–1.16
		Richest [ref]	1	---	---	1	---	---
	*Health insurance coverage*							
		Covered [ref]	1	---	---	1	---	---
		Not covered	1.35	<0.001	1.32–1.38	1.22	<0.001	1.18–1.27
	*Distance to health facility *							
		No problem [ref]	1	---	---	1	---	---
		Big problem	1.69	<0.001	1.65–1.73	1.25	<0.001	1.18–1.32
		Not a big problem	1.41	<0.001	1.38–1.44	1.12	<0.001	1.08–1.17
	*Getting money for treatment *							
		No problem [ref]	1	---	---	1	---	---
		Big problem	1.43	<0.001	1.39–1.46	0.68	<0.001	0.64–0.72
		Not a big problem	1.33	<0.001	1.29–1.36	0.88	<0.001	0.84–0.92
	*Receiving permission to seek medical help*							
		No problem [ref]	1	---	---	1	---	---
		Big problem	1.75	<0.001	1.71–1.80	1.57	<0.001	0.64–0.72
		Not a big problem	1.37	<0.001	1.34–1.40	1.33	<0.001	0.84–0.92
**Need factors**	*Pregnancy intention*							
		Wanted [ref]	1	---	---	1	---	---
		Not wanted/Wanted later	1.55	<0.001	1.50–1.61	1.22	<0.001	1.15–1.28
	*Birth order*							
		1st [ref]	1	---	---	1	---	---
		2nd– 3rd	1.35	<0.001	1.32–1.38	1.32	<0.001	1.27–1.37
		4th– 5th	2.61	<0.001	2.53–2.71	1.79	<0.001	1.69–1.90
		≥6^th^	4.21	<0.001	3.94–4.50	2.43	<0.001	2.20–2.69

a: Regions defined by NFHS-5 report [25].

Both type of residence and region were significantly associated with ANC utilization. Women living in rural India had higher odds inadequate ANC visits compared to their urban counterparts (aOR = 1.12, 95% CI: 1.06–1.18, p<0.001). Women residing outside of Southern India were also more likely to underutilize antenatal care visits. Specifically, those from Northeastern and Central states had the highest odds of inadequate ANC visits compared to women from Southern states with adjusted odd ratios of 3.06 (CI: 2.81–3.34, p<0.001) and 2.92 (CI: 2.73–3.12, p<0.001), respectively.

Among the predisposing factors included in our multivariate analysis, education level and caste were significantly associated with antenatal care visits. Odds of ANC underutilization increased with lower levels of formal education. Women reporting no education had 1.76 (CI: 1.65–1.88, p<0.001) times the adjusted odds of ANC underutilization compared to the reference group of women who had attended higher education. Those belonging to scheduled castes and other backward classes also had higher adjusted odds of inadequate ANC visits compared to women who identified as not belonging to a scheduled caste/tribe or other backward class.

All explanatory variables identified as need or enabling factors were significantly associated with ANC utilization. Women belonging to poorer households had higher odds of inadequate ANC visits compared to women from wealthiest households. For example, women from the poorest households had 1.69 (CI: 1.57–1.81, p<0.001) times the adjusted odds of inadequate ANC compared to those from the wealthiest households. Those not covered by health insurance were also more likely to underutilize antenatal care visits compared to women with health insurance coverage. Notably, women who reporting “no problem” with getting money for medical treatment had higher odds of inadequate ANC visits compared to women who this as a problem (either a “big problem” or “not a big problem”). Women who reported problems with distance to health facilities or with receiving permission to seek medical services from a health profession were more likely to underutilize ANC.

Odds of ANC underutilization increased with increasing birth order. Women giving birth to their sixth child had 2.59 (CI 2.34–2.86, p<0.001) times the adjusted odds of inadequate ANC visits compared to women having their first child. Women giving birth to their fourth or fifth child had 1.84 (CI: 1.75–1.95, p<0.001) times the adjusted odds of inadequate ANC visits compared with the reference group. Women giving birth for the second or third time had 1.27 (CI: 1.22–1.31, p<0.001) the adjusted odds of inadequate ANC visits compared with the reference group. Pregnancy intention was also associated with antenatal care utilization. Those who indicated their last child was not wanted at time of pregnancy had higher odds ANC underutilization compared to those who wanted their child (aOR = 1.22, 95% CI: 1.15–1.28, p<0.001).

## Discussion

Findings from this study demonstrate that 59.3% of pregnant women in India had four of more ANC visits–a steady increase from 51.7% and 32.5% as demonstrated in the NFHS-4 (2015–2016) and NFHS-3 (2005–2006), respectively [26, 27]. Despite this positive development, there is cause for concern. Notably, while India outperforms South Asia as a whole, the percentage of Indian women receiving adequate ANC visits is still below the global average of 66.3% [28]. Furthermore, our analysis reveals a continuity in the groups of women at highest risk for inadequate ANC [10, 19].

Consistent with previous research, pregnant women from Northeastern and Central states continue to be at highest risk for inadequate ANC visits [18–20]. Regional disparities in ANC visits are perhaps partly attributable to variations in overall development, which likely contribute to gaps in healthcare access [29, 30]. Many Southern states enjoy higher per-capita incomes, better access to basic household amenities (i.e. drinking water, electricity, etc.), superior healthcare infrastructure, and greater availability of physicians [31].

Women living in comparatively rural areas also had higher odds of underutilizing antenatal care services despite the decades-long existence of India’s National Rural Health Mission (NRHM), which was implemented, in part, to address rural-urban gaps in high quality healthcare access. Since the NRHM’s initiation, studies have shown that residents from relatively remote communities remain less exposed to skilled healthcare professionals [32]. Limited physical connectivity and poor referral systems are a few of the many challenges rural women face when trying to access established healthcare providers [33, 34].

Our findings indicate significant disparities in antenatal care utilization across maternal educational attainment and household wealth. Women with no formal education and those from the poorest households were at highest risk of inadequate ANC. This is in line with previous evidence around socioeconomic inequalities in maternal health service utilization, both in India and South Asia [1, 9, 19, 35–37]. For instance, Goel and colleagues’ 2015 study analyzing NFHS-3 data concluded that wealth index was the leading key independent determinant for three or more ANC visits received while maternal literacy was the leading independent key determinant for early antenatal registration [35]. Similarly, a recent study based on utilization of maternal and child health services in Bangladesh underscores socioeconomic status as a primary driver of completion of continuum of care [38]. Whilst educational and wealth inequalities have been improving since the early 2000s, socioeconomic trends in antenatal care service utilization highlight ingrained inequalities in maternal health across South Asia [1, 9, 34, 39–41].

Pregnancy intention and birth order were also significantly associated with antenatal care utilization in our study. Women who did not want their pregnancy or wanted it later had a higher odds of ANC underutilization. These finding are consistent with trends noted globally–in both developed and developing countries [42, 43]. Prior research has demonstrated that women with unintended pregnancy are more likely to delay their initiation of antenatal care services [42].

Our study reiterates the continued importance of empowering women and improving their access to healthcare–particularly those living in rural settings, those from the poorest households, and those with little formal education. In the long term, poverty alleviation, equitable investment in infrastructure, and education should be pursued as priority areas to achieve more equitable maternal health service utilization [9, 19, 38, 44–46]. In the short-term, interventions such as group antenatal care and mHealth initiatives (using mobile phones applications to help connect women with information and services)–have shown some promise in LMICs and should be considered [47–52]. India can also adapt lessons learned from community-based outreach interventions targeting high-risk groups [36].

### Strengths & limitations

Our analysis used data from the most recent iteration of a nationally representative survey sample, which is a noteworthy strength. We also explored the interaction between ANC utilization and a diverse array of explanatory variables. However, our study has many limitations. Notably, responses to NFHS-5 were self-reported, introducing possible recall bias. Our definition of adequate ANC utilization does not reflect the WHO’s most recent guidelines, which recommend eight or more ANC visits [11]. Furthermore, while we focused on antenatal care visits, our study did not consider other recommended health behaviors during the antenatal period such as iron supplementation and tetanus toxoid vaccination.

We recommend additional research exploring quality of care during ANC visits–i.e. coverage and adequacy, timeliness of visits, clinical competency of recommendations made, and nature of patient-provider communication [17]. Qualitative analysis exploring the experiences of women from different sociodemographic groups would help policymakers better understand the challenges that high-risk groups face when seeking antenatal care. Additional research utilizing WHO’s ANC monitoring framework to look beyond ANC visits and investigate barriers to positive pregnancy experience is also needed.

## Abbreviations

aOR: Adjusted odds ratio
ANC: Antenatal care
CI: Confidence interval
DHS: Demographic and Health Survey
IIPS: International Institute for Population Sciences
LMIC: Low- and middle-income country
MMR: Maternal Mortality Ratio
NMR: Neonatal Mortality Ratio
NFHS: National Family Health Survey
NRHM: National Rural Health Mission
OR: Odds ratio
SDGs: Sustainable Development Goals
WHO: World Health Organization
